# Evaluation of an Intraoral Camera with an AI-Based Application for the Detection of Gingivitis

**DOI:** 10.3390/jcm14155580

**Published:** 2025-08-07

**Authors:** Cécile Ehrensperger, Philipp Körner, Leonardo Svellenti, Thomas Attin, Philipp Sahrmann

**Affiliations:** 1 Clinic of Conservative and Preventive Dentistry, Center for Dental Medicine, University of Zurich, Plattenstrasse 11, 8032 Zurich, Switzerland; 2 Department of Periodontology, Endodontology and Cariology, University Center for Dental Medicine Basel UZB, University of Basel, Mattenstrasse 40, 4058 Basel, Switzerland

**Keywords:** gingivitis, oral health assessment, camera-based diagnostics, digital dentistry, dental imaging, deep learning, AI

## Abstract

**Objective:** With a global prevalence ranging from 50% to 100%, gingivitis is considered the most common oral disease in adults worldwide. It is characterized by clinical signs of inflammation, such as redness, swelling and bleeding, on gentle probing. Although it is considered a milder form of periodontal disease, gingivitis plays an important role in overall oral health. Early detection and treatment are essential to prevent progression to more severe conditions. Typically, diagnosis is performed by dental professionals, as individuals are often unable to accurately assess whether they are affected. Therefore, the aim of the present study was to determine to what degree gingivitis is visually detectable by an easy-to-use camera-based application. **Materials and methods:** Standardized intraoral photographs were taken using a specialized intraoral camera and processed using a custom-developed filter based on a machine-learning algorithm. The latter was trained to highlight areas suggestive of gingivitis. A total of 110 participants were enrolled through ad hoc sampling, resulting in 320 assessable test sites. A dentist provided two reference standards: the clinical diagnosis based on bleeding on probing of the periodontal sulcus (BOP) and an independent visual assessment of the same images. Agreement between diagnostic methods was measured using Cohen’s kappa statistic. **Results:** The agreement between the application’s output and the BOP-based clinical diagnosis was low, with a kappa value of 0.055 [*p* = 0.010]. Similarly, the dentist’s visual assessment of clinical photos showed low agreement with BOP, with a kappa value of 0.087 [*p* < 0.001]. In contrast, the agreement between the application and the dentist’s photo-based evaluations was higher, with a kappa value of 0.280 [*p* < 0.001]. **Conclusions:** In its current form, the camera-based application is not able to reliably detect gingivitis. The low level of agreement between dentists’ visual assessments and the clinical gold standard highlights that gingivitis is difficult to identify merely visually. These results underscore the need to refine visual diagnostic approaches further, which could support future self-assessment or remote screening applications.

## 1. Introduction

Oral health is an integral component of overall health and well-being. The perceived condition of one’s oral cavity significantly impacts daily functioning and is commonly assessed through the concept of oral-health-related quality of life (OHRQoL). This measures how oral diseases and conditions affect a person’s physical, emotional and social well-being [1,2]. Numerous studies have shown that OHRQoL can be substantially impaired by oral diseases and their consequences, such as pain, discomfort, esthetic concerns and functional limitations [3].

Among the most prevalent oral diseases globally is dental caries, affecting individuals across all age groups [4]. Periodontal diseases are also widespread and represent a major public health burden. While advanced oral conditions often lead individuals to seek professional care, many diseases frequently progress without noticeable symptoms [5].

Gingivitis is the minor form of periodontal disease, which is one of the most common oral diseases and conditions in adults [6]. It is also the most prevalent form of periodontal disease in children and adolescents [7] due to poor oral hygiene and hormonal changes. 

Periodontitis, the severe form of periodontal disease, is a non-communicable chronic inflammatory disease that affects all parts of the periodontium and causes largely irreversible damage. Advanced forms of periodontitis are characterized by significant destruction of the periodontal ligament and marginal alveolar bone, resulting in tooth mobility, tooth migration, gingival recession, and finally, tooth loss [8]. Since the respective inflammation is primarily chronic and painless, periodontitis is often described as a “silent” disease [9].

The inflammation is triggered by components from microbial plaque accumulating on the non-shedding tooth surfaces at or near the gingival sulcus [10]. Inflammation is clinically manifested by the main features of redness, swelling and bleeding on periodontal probing. If untreated, enhanced pocket depths due to inflammatory destruction of the periodontal tissues are found, and the disease is then called periodontitis [11]. In gingivitis, the redness of the gingival margin arises partly from the dilatation and enhanced perfusion of capillary blood vessels in the subepithelial connective tissue [12]. 

Bleeding on brushing and bleeding on interdental cleaning have been suggested as identifying features for patients. Since self-diagnosis remains difficult, periodontal diseases are usually detected at a late stage, and proper localization and extent can only be diagnosed by trained dental professionals. For gingivitis, the clinical parameter of bleeding on gentle probing is the accepted gold standard [11]. 

Regular dental appointments for early detection are therefore essential for prevention and (if needed) adequate treatment of this disease. Nevertheless, many individuals, especially younger adults, do not regularly seek dental care and tend to underestimate the early signs of gingival inflammation. The asymptomatic nature of mild gingivitis, combined with low awareness of its potential long-term consequences, often results in delayed diagnosis and treatment. Given the high prevalence and potential for progression into irreversible disease, the demand for easily accessible, low-barrier diagnostic support systems is growing.

For self-diagnosis of gingivitis that can easily be performed by the patient themselves, the first attempts were proposed in 1992 [13]. Gingivitis was examined using photographs for its characteristic redness and swelling of the gums. Over the years, more studies based on clinical photos of the gingiva and additional compatible software like Serif photo plus-6 [14], Image Pro Plus software (V4.0; Media Cybernetics, Silver Spring, MD, USA) [15] had been developed. All respective studies are based on measuring redness and swelling before and after gingivitis treatment. The advancement in the development of digital technology also enabled the exploration of the tissues’ volumes via more refined imaging techniques, such as 3D scans (3Shape A/S, Copenhagen, Denmark) [16].

To detect oral diseases at an early stage and to eventually treat them via specific home care treatment, recently developed computer applications may be of benefit. For example, apps to improve toothbrushing technique have been invented. Examples of these include the Oral-B app and the Philips Sonicare app, which are both available in the App Store and Google Play Store. Since 2021, another provider aimed for diagnosis and developed a filter application for an intraoral camera, which allows patients to perform a self-check on their respective oral hygiene and gingivitis status. The application claims to recognize discolorations, calculus and gingivitis [17].

This intraoral camera-based filter application is based on a supervised machine-learning algorithm, which is based on the principles of artificial intelligence (AI) and machine learning. In contrast to the studies mentioned earlier that relied on static photographs and manual or semi-automated analysis of gingival inflammation, this AI-based system enables automated and scalable detection by identifying complex visual patterns associated with gingivitis. For the present instrument, this means that the algorithm is trained with hundreds of thousands of images. These had previously been assessed for gingivitis by experienced dentists, based on the main characteristics of red or swollen gums in the vicinity of calculus. The neural network classifies each pixel with a so-called segmentation algorithm [18]. This type of gadget is primarily aimed at a target group of young adults with interest in both easy computerized applications and their oral hygiene.

Despite many studies involving photographic assessment of gingivitis [13,14,15,16], a proper validation of an app-based detection feature for redness and swelling is still missing. Therefore, the fact that only the surface but no deeper tissue layers can be assessed by an optical system is a relevant limitation in the detection of periodontal diseases so far. Accordingly, the proof of correlation with gingivitis diagnosis using merely optical means like photo assessment or filter-based application is still missing.

Therefore, the aim of the study was to determine whether an intraoral camera-based application can visually detect gingivitis. Accordingly, we hypothesized that gingivitis could be reliably detected either by an app using a filter application or by a dentist based solely on optical features.

## 2. Materials and Methods

### 2.1. Study Design and Clinical Assessment

The study was designed as clinical data collection of primary data via a single examination. The study plan was approved in advance by the ethics committee of the University of Zurich. Data were collected from subjects at the Center of Dental Medicine Zurich (ZZM, University of Zurich). Study participation was strictly voluntary and adhered to the principles of the Declaration of Helsinki [19].

The inclusion criteria comprised minimum age of 18 years and understanding of the patient information in German. The exclusion criteria included optically detectable amalgam tattoos of the gingival margin, ethnic pigmentation of the gingiva and gingival hyperplasia or other mucosal changes in the test area and edentulous patients or patients with necrotizing ulcerative gingivitis/necrotizing ulcerative periodontitis (NUG/NUP) or severe periodontitis (probing depth ≥ 6 mm/already being treated by a periodontist). The participants were determined by an ad hoc sample, i.e., potential subjects who met the inclusion criteria were randomly asked at the clinic (Clinic of Preventive and Conservative Dentistry, ZZM, University of Zurich) or outside the ZZM, asking friends and family whether they would like to take part in the study. 

To assess the intraoral situation of the marginal gingiva, in each patient, three specific test sites were analyzed. The teeth and surrounding gingiva were photographed using an intraoral camera [17]. The device is equipped with a 2-megapixel CMOS imaging sensor and provides a resolution of 1920 × 1080 pixels. The focal length ranges from 10 to 20 mm, allowing for close-up imaging of oral structures. Eight adjustable LEDs surrounding the lens ensure consistent illumination, while the 70° viewing angle offers an optimal field of view. The waterproof lens (IP67 rating) and wireless connectivity (IEEE 802.11 b/g/n, up to 10 m range) further supported usability and hygiene during clinical application. These camera settings were always the same, in accordance with our clinic’s standard operating procedures. After image acquisition, the clinical bleeding on probing was evaluated.

The intraoral images were anonymized and assessed for gingivitis and calculus on a 10.2-inch iPad (Apple, Cupertino, CA, USA) twice: in one group, the photos were assessed by a filter application [17], and in the other group, the pictures were evaluated by one experienced dentist, who was unaware of the clinical parameter of bleeding on probing. In both groups, the presence or absence of gingivitis was recorded dichotomously. In order to assess the quality of the assessment, in the second step, these results were compared with the clinical gold standard (BOP). Likewise, agreement between the groups was assessed.

In an additional analysis, the presence of dental calculus was compared with the presence of BOP-positive findings to assess whether gingivitis occurred more frequently in the presence of calculus.

### 2.2. Clinical Photograph

The gingiva around selected teeth was photographed orthogonally using an intraoral camera [17] from a distance of approximately 2 cm. Images were taken directly, i.e., without the use of a mirror, in the regions of tooth 16 (vestibular), tooth 36 (lingual) and tooth 41 (lingual), where gingivitis was most likely to occur. In cases where the image was blurred or the tooth was not fully captured, the photograph was repeated. A maximum of three attempts were permitted. If no satisfactory image could be obtained after the third attempt, the final image was accepted, as further improvement was deemed unlikely.

Afterward, bleeding on probing was clinically determined 10 s after swiping the sulcus with a standard periodontal probe with a diameter of 0.5 mm and an application force of 0.25 N and recorded for later comparison. Both imaging and BOP on the same subject were performed by the same trained operator. By contrast, the examiner assessing the images was unaware of the BOP findings to ensure blinded evaluation.

### 2.3. Functionality of the Camera and Its Application

**Figure 1:**
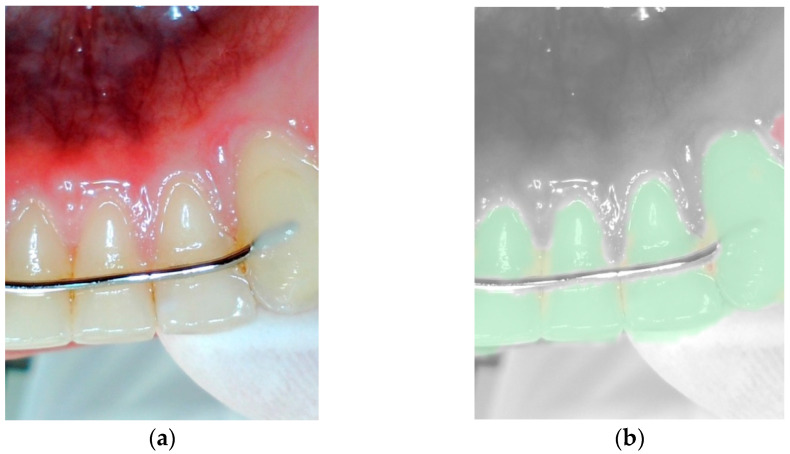
Image with healthy gingiva, without (**a**) and with (**b**) the filter application.

**Figure 2:**
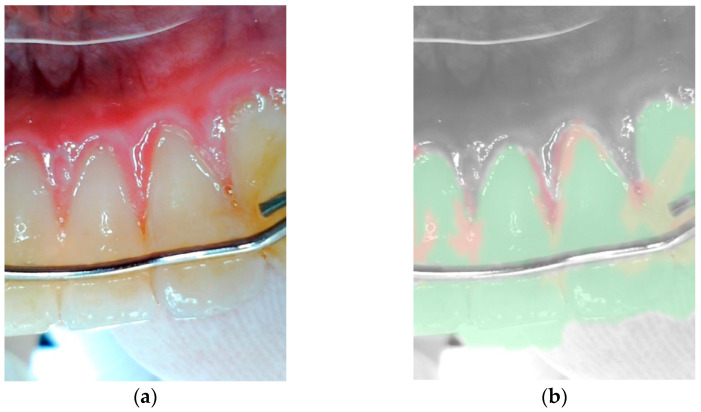
Image with gingivitis in regio 31 in the distal part and regio 32 in the mesial part of the papillae, without (**a**) and with (**b**) the filter application.

The intraoral camera [17] was wirelessly connected to a dedicated filter application [17] on a 10.2-inch iPad (Apple, Cupertino, CA, USA) via Bluetooth. The captured images were displayed directly within the application interface. Upon activation of an integrated analysis filter, the software provided a visual overlay enabling the identification of extrinsic discoloration, dental calculus and potential signs of gingivitis. In this diagnostic mode, tooth surfaces were visualized in distinct colors to indicate their condition: green signaled clean and healthy areas; orange highlighted the presence of discoloration or tartar build-up; and red indicated potential signs of gingivitis or inflammation. These red-marked regions were systematically identified, read off the display and recorded as part of the overall clinical evaluation process (see Figure 1 and Figure 2).

### 2.4. Statistics

Based on the assumption of a difference of 10% (effect size) for the gingivitis diagnosis by either the intraoral camera or the clinical examination by the dentist and a power of 90% with a significance level of 5%, 266 test sites with 3 test sites per subject were calculated as the minimum number needed to show statistical difference. Accordingly, 3 sites per patient were assessed. For agreement between the optical assessment by either the dentist or the application, kappa values and percentage of correlation were calculated. Association between the results of the different testing groups were tested by Pearson’s Chi-squared test. Generally, the level of significance was set at 0.05. All tests were calculated with SPSS (Vs. 28.0.1.1 (14), IBM Corp., Armonk, NY, USA).

### 2.5. Ethics

According to the Swiss Human Research Act (HRA), this study requires submission to the Ethics Committee Zurich. It falls under the scope of the Human Research Ordinance (HRO), excluding clinical trials, and involves data collection from human participants classified as risk category A. The ethics application was approved by the Ethics Committee Zurich in 2022. The BASEC ID for this study is 2021-02387.

## 3. Results

The present study was conducted as a single examination from May to the end of August 2022 at the Clinic for Conservative and Preventive Dentistry at the Center for Dental Medicine of University of Zurich. The study assessed gingivitis in 110 subjects.

Due to the one-time examination, there were no dropouts. Participants ranged in age from 18 to 78 years, with most subjects between 20 and 29 years old. Three designated test sites were analyzed per subject. Of the 330 initially targeted sites, 320 were ultimately included in the final evaluation. While all subjects remained in the study, 10 sites had to be excluded. Specifically, three sites were excluded due to ethnic pigmentation of the gingiva (two at tooth 16, one at tooth 41), two sites due to extensive amalgam restorations (both at tooth 36) and one site because of the presence of an orthodontic bracket (at tooth 16). In four cases, assessment was not possible, as the corresponding test teeth were missing (all four at tooth 36). Of the 320 test sites evaluated for bleeding on probing, 118 tested positive: 33 at tooth 16, 43 at tooth 36 and 42 at tooth 41. The filter-based assessment identified 125 sites as indicative of gingivitis: 33 at tooth 16, 32 at tooth 36 and 60 at tooth 41. In comparison, the clinical evaluation by the dentist classified a total of 89 sites as gingivitis: 34 at tooth 16, 19 at tooth 36 and 36 at tooth 41.

### Analysis

The agreement between gingivitis diagnosis via filter application and the clinical findings of gingivitis (BOP) was expressed by a kappa value of 0.055 as statistically significant agreement. The corresponding diagnostic accuracy metrics were as follows: sensitivity 66.3%, specificity 48.3% and positive predictive value 68.7%. If the results were assessed separately, the kappa value was 0.036 at tooth 16, 0.114 at tooth 36 and 0.247 at tooth 41, respectively (see Table 1).

The total kappa value for the inter-group correlation of photo evaluation by the dentist and the clinical finding of gingivitis was 0.087, with a significant agreement of *p* < 0.001. To complement the kappa statistic, diagnostic accuracy metrics were calculated: the sensitivity was 79.7%, specificity 40.7% and positive predictive value 69.7%. The kappa values for individual teeth were 0.196 for tooth 16, 0.265 for tooth 36 and 0.204 for tooth 41. The gingiva was classified as healthy by the dentist in case of doubt, which led to an increased number of false negative results (see Table 2).

To determine the extent to which the results of the filter-based application differ from the dentist’s visual assessment of the photographs, Table 3 presents a comparative analysis. All data were evaluated based on whether gingivitis was visually detected by either the filter application or the dentist. The total kappa value was 0.280, indicating a statistically significant agreement (*p* < 0.001), which is higher than the agreement between either the dentist and the clinical reference or the app and the clinical reference. When examining individual teeth, the kappa values were as follows: 0.153 for tooth 16, 0.262 for tooth 36 and 0.361 for tooth 41. The agreement for teeth 36 and 41 was statistically significant, whereas the value for tooth 16 did not reach statistical significance (*p* = 0.114). In numerical terms, the assessments were concordant in 212 out of 320 test sites. At the time of data analysis, no comparison with the Bleeding on Probing index had been performed.

Finally, the kappa value for the correlation between calculus and BOP was evaluated. Since calculus was mainly located on test tooth 41, only the values of this tooth were assessed. No calculus was detected on test tooth 36 (lingual), and only two instances were found on test tooth 16 (vestibular). Due to the limited relevance of these findings, they were not included in further analysis. Among the 109 test teeth analyzed, calculus was detected on 61. The kappa value was 0.089 and did not show a significant association with 0.323.

## 4. Discussion

The aim of the study was to assess whether gingivitis may be diagnosed by a filter application and an intraoral camera operating solely on an optical base. Optical assessment of the respective photos by a dentist on the one hand and clinical assessment on the other hand served as a comparison and validation. Based on the data of the present assessment, it can be concluded that there is only limited agreement between the app and the clinical finding of gingivitis (see Table 1). The diagnosis by the dentist for recognizing gingivitis based on the images, however, is low to a similar degree (see Table 2). Therefore, Table 1 and Table 2 show kappa values that reflect no agreement beyond chance. This indicates that even trained dental professionals are unable to reliably detect gingivitis through purely optical evaluation of intraoral photographs. Therefore, our hypothesis that gingivitis can be detected by an app and its filter application or a dentist based on purely optical features must be rejected.

Many aspects make it difficult to assess whether gingivitis is present. During the investigation, testing for BOP was sometimes hampered in cases of abundant presence of calculus because access to the sulcus is sometimes limited. The analysis shows that BOP and calculus do not exhibit a significant association. It is difficult to determine whether this is due to limitations in the study, such as the challenge of accurate validation in the presence of calculus, or whether there truly is no association. This methodological limitation may have affected the validity of BOP as a diagnostic reference and potentially contributed to false negative results.

In the present study, the operator was well trained to handle the camera. Nevertheless, technical challenges were encountered. Achieving the correct distance from the tooth of interest was essential to obtain focused, diagnostically usable images. Particularly in the posterior molar area, this was sometimes difficult to achieve due to limitation by cheek or tongue. Saliva with air bubbles at the gingival margin posed a considerable challenge in acquiring diagnostically adequate images. Additionally, intermittent lens fogging may have further compromised the quality of the intraoral photographs. 

Furthermore, there are also some inherent limitations of the application. To prevent a potential bias, amalgam tattoos, ethnic pigmentation of the gingiva and gingival hyperplasia had to be excluded from the study because the filter application had not been trained sufficiently to cope with these changes. Other problems were noted with the recognition of teeth with large metal restorations like amalgam fillings, gold crowns or when orthodontic brackets were attached on the teeth.

Clinically, gingivitis is diagnosed by the dentist based on a comprehensive set of symptoms. Optically, redness, the texture of the gingiva (stippling, swelling), spontaneous and provoked bleeding may be used. While the latter is a well-assessed and scientifically approved way to clinically assess gingivitis when correct pressure is used [20], assessment of a purely optical analysis has rarely been performed. Furthermore, the diagnostic value of intraoral photographs is inherently limited by lighting conditions, such as shadows or overexposure, especially under non-standardized settings. Nevertheless, the agreement between the dentist and the filter-based application was higher than with the clinical gold standard (Table 3), suggesting some consistency between both visual assessments. The kappa value demonstrated a fair agreement between the assessed methods.

Notably, the dentist tended to classify ambiguous cases as healthy, which likely increased specificity at the expense of sensitivity and contributed to false negative results. Therefore, the discrepancy between the results from the filter application and the dentist may have been distorted. A closer look at the raw data supports this assumption: among a total of 320 images, only 89 were diagnosed as showing signs of gingivitis by the dentist, while the filter application identified 125 cases. Optimizing both the camera system and the filter application may help reduce the respective bias and improve diagnostic performance to a certain extent. The filter, which is based on deep-learning algorithms, might also show some potential for improvements. Artificial intelligence is already being applied and actively investigated in several fields of dentistry. In endodontology, artificial intelligence is helping to study the anatomy of the root canal system, forecasting the viability of stem cells of the dental pulp, measuring working lengths, pinpointing root fractures and periapical lesions and forecasting the success of retreatment procedure [21]. In cariology, artificial intelligence is successful in detecting caries lesions of varying radiological extent on bitewings [22]. In the research of orthodontics, artificial intelligence has been applied in the analysis of clinical imagery, such as automated landmark detection in lateral cephalograms and evaluation of intraoral scans or photographic data. Furthermore, artificial intelligence supports orthodontists with treatment decisions, such as the need for orthognathic surgery or orthognathic tooth extractions [23]. One of the most recent studies demonstrates that AI can also be effective in patient education. It compares the effectiveness of AI-generated educational materials with traditional methods in improving patient understanding and reducing anxiety prior to dental procedures [24]. Another contemporary study supports dentists in treatment decision making by assisting in the detection of periapical radiolucencies on panoramic radiographs [25].

Since the dentist’s image-based assessment did not show a high agreement with the clinical gold standard, visual diagnosis of gingivitis from photographs is likely to show fundamental limitations, regardless of human or artificial interpretation. This raises the question of whether improvements in the current filter algorithm alone are sufficient to substantially increase diagnostic reliability. Alternative strategies, such as the combination of visual, tactile and biochemical data, may be options to optimize both the device and the application. 

Future developmental steps could involve the integration of additional diagnostic modalities. One promising approach is fluorescence-based detection of gingival biomarkers. A study by Rana et al. [26], which followed a similar study design, used light with a wavelength ranging from 405 to 450 nm to stimulate fluorescence of porphyrin-producing bacteria associated with periodontal inflammation. Using a specialized oral imaging device (ACTEON, North America), the emitted fluorescence signals were detected, and healthy tissue could be disclosed by its characteristic coloration. The study demonstrated that the method could differentiate between inflamed and healthy gingiva with an accuracy of more than 50%. Other studies have explored visual gingivitis detection using standardized photographic comparisons before and after professional tooth cleaning [14,15]. However, these studies often involved patients with clearly visible plaque and calculus, which makes diagnosis easier and could lead to an overestimation of diagnostic performance. In contrast, the present study included subjects with mild and therefore less clinically evident gingival inflammation. Since the age of participants primarily ranged between 20 and 30 years and they exhibited above-average oral hygiene, often lacking visible signs, such as plaque or swelling, the presence of gingivitis was not obvious. Therefore, the identification of gingivitis represented a more challenging diagnostic task. The relatively young age of the study population was mainly due to the limited willingness of older individuals to participate. Then, older volunteers often presented with amalgam restorations, increased STI (>6) in the molar area or even missing teeth, which led to the exclusion of certain sites from evaluation. A more balanced age distribution among participants—and consequently, a broader range of gingivitis severity—might have enhanced the study’s generalizability. The generalizability was further limited by the exclusion of pigmentation, as this occurs predominantly in certain ethnicities due to their darker skin color. 

It would also be of interest if, at the time of clinical photo acquisition and BOP assessment, two independent examiners performed the evaluation. This would allow for an assessment of inter-examiner variability and the extent to which diagnostic outcomes may differ between clinicians. Furthermore, conducting the study using an improved version of the filter application would be very valuable. At the same time, images of people in more age groups should be evaluated, with a clear diagnosis of gingivitis. This would likely improve the generalizability of the results and enable more robust, broadly applicable conclusions to be drawn. Additionally, further training and refinement of the AI model could reveal whether diagnostic accuracy and overall performance could be significantly enhanced, thereby increasing its clinical utility.

The integration of artificial intelligence into oral care is an expanding and increasingly accessible field. Numerous digital health technologies are being developed to enhance preventive care, improve patient adherence and support remote monitoring. For example, smart toothbrushing systems are now capable of analyzing brushing patterns, detecting missed areas and providing real-time feedback through app-based interfaces [27]. These systems often use similar algorithmic principles to the gingivitis detection application described in this study.

In the future, the combination of different AI-supported tools—such as intraoral imaging, brushing analysis and symptom tracking—could provide users with a more comprehensive picture of their oral health and serve as a valuable adjunct in teledentistry and preventive care. However, the present results underline that current AI-based applications, particularly those relying solely on visual information, still face significant limitations in diagnostic accuracy and should be regarded (in future) as complementary tools rather than replacements for clinical examination.

## 5. Conclusions

The present study demonstrates that the visual detection of gingivitis using the present intraoral camera is unreliable. Neither the application of an AI-based filter nor the dentist’s visual assessment achieved a satisfying level of agreement with the clinical reference standard. This highlights the diagnostic challenges of relying solely on optical features without clinical probing.

## Figures and Tables

**Table 1:** Correlation of clinically detected gingivitis and diagnosis by the app.

	N	Kappa Value	Asymptomatic Standard Errors	Significance *
Tooth 16	107	0.036	0.098	0.709
Tooth 36	104	0.114	0.096	0.232
Tooth 41	109	0.247	0.087	0.006
Teeth 16, 36, 41	320	0.055	0.021	0.010

* Pearson’s Chi-squared test, significance value < 0.05.

**Table 2:** Correlation of clinically detected gingivitis and visual diagnosis from the photo by the dentist.

	N	Kappa Value	Asymptomatic Standard Errors	Significance *
Tooth 16	107	0.196	0.099	0.042
Tooth 36	104	0.265	0.086	0.002
Tooth 41	109	0.204	0.095	0.032
Teeth 16, 36, 41	320	0.087	0.022	<0.001

* Pearson’s Chi-squared test, significance value < 0.05.

**Table 3:** Correlation between app-based gingivitis diagnosis and visual diagnosis from the photo by the dentist.

	N	Kappa Value	Asymptomatic Standard Errors	Significance *
Tooth 16	107	0.153	0.099	0.114
Tooth 36	104	0.262	0.100	0.005
Tooth 41	109	0.361	0.079	<0.001
Teeth 16, 36, 41	320	0.280	0.054	<0.001

* Pearson’s Chi-squared test, significance value < 0.05.

## Data Availability

The data presented in this study are available upon request from the first author. The data are not publicly available due to privacy concerns.
